# An interesting case of serotonin syndrome precipitated by escitalopram

**DOI:** 10.4103/0253-7613.71899

**Published:** 2010-12

**Authors:** Debasish Sanyal, Suddhendu Chakraborty, Ranjan Bhattacharyya

**Affiliations:** Department of Psychiatry, Calcutta National Medical College, Kolkata, India

**Keywords:** Escitalopram, serotonin, syndrome, therapeutic dose

## Abstract

Serotonin syndrome is a known entity, which occurs with multiple drugs acting on serotonergic receptors. A 73-year-old lady presented with a history of agitation, altered sensorium, and autonomic hyperactivity after starting escitalopram on therapeutic dosage for her depressive syndrome who was on selegiline for her parkinsonism. This syndrome with therapeutic dose escitalopram warrants the careful and judicious use of the drug especially with other serotonergic drugs, so that this serious medical complication can be avoided.

## Introduction

Serotonin syndrome has been regarded as a rare but potentially fatal syndrome associated with the introduction and an increase in dose of a serotonergic agent. It is commonly characterized by agitation, tremors, shivering, diarrhea, hyperreflexia, hyperthermia, ataxia, and altered sensorium. Incidence of this syndrome is less than 1% as most of the cases remain unreported. Serotonin syndrome is common in drug combinations involving selective serotonin reuptake inhibitors (SSRIs) and many other drugs such as amphetamine, Monoamine oxidase inhibitors (MAOI), tricyclic antidepressants, lithium, buspirone, dextromethorphan, linezolid, tramadol, etc. It rarely occurs in therapeutic doses.[1–3]

## Case Report

A 73-year-old lady presented to the psychiatry outpatient department of the tertiary care hospital in November 2009 with complaints of restlessness and chest discomfort for the last 3 days. History as obtained from the informant (son) revealed that she had been attending a neurologist for treatment of parkinsonism for which she had been given selegiline (5 mg per day).Thereafter, she became sluggish in her activities, lost interest in pleasurable activities, and developed low mood for which she consulted a psychiatrist who prescribed her escitalopram (10 mg per day) to address her depressive symptoms. Her present complaints started 1 week after initiating the new medicine. There was no history of any substance abuse. The patient did not take any other medicines during this period. The onset and progression of the disease was rapid.

She was admitted to medicine department initially and shifted to the ICCU immediately. Detailed clinical evaluation showed that the patient was having agitated behavior and restlessness. She was shivering and sweating profusely and was having diarrhea. Her oral temperature was found to be 102°F. She was having tremor, ataxia, and hyperreflexia (which were found to be more in the lower limbs than the upper limbs). She also had mydriasis, tachycardia, and hypotension. Mental status examination showed that the patient was conscious confused, with features of intense anxiety, agitation, and restlessness. Her routine blood counts, blood glucose level estimation, urea, creatinine, serum electrolytes, liver function test, and creatinine phosphokinase levels were within normal limits. The cardiac enzymes including troponin T levels were inconclusive while the electrocardiogram showed only a few nonspecific ST-T changes along with sinus tachycardia. CT scan of brain showed diffuse cerebral atrophy, and a few ischemic changes in the periventricular regions. Her urinary myoglobin level was within normal range. Her body weight was 60 kg.

The patient was initially given intravenous fluids to correct dehydration and supportive management with immediate discontinuation of all the serotonergic drugs. She was prescribed with tablet cyproheptadine, initially 4 mg orally at 2–4 hourly intervals up to 30 mg/day for the first day. On the next day, the patient was symptomatically better and her vitals became stable. The patient was discharged after 1 week and reported for follow-up in the psychiatry OPD where she was put on low dose quetiapine for its calming effect and safety in parkinsonism.

## Discussion

Serotonin syndrome usually occurs with nonselective MAO inhibitors due to MAO-A inhibition. However, there are few cases which have highlighted this possibility with MAO-B inhibitor also.[4,5] As was evident from the case profile, the patient met the Sternbach’s diagnostic criteria with five of the 10 symptoms mentioned in criterion 4.[6] The assessment by Naranjo’s algorithm was done which revealed a score 7 which suggests a probable (ADR) adverse drug reaction.[7] Assessment of preventability of the ADR was done based on modified Schumock and Thornton scale. Most of the ADRs belonged to the category of not preventable. However, the more common reactions like diarrhea belonged to the category of probably preventable.[8]

While initial clinical evaluation and investigations ruled out probable infective and metabolic causes, the rapid onset and progression of the disease and the absence of concurrent antipsychotic drug usage in this case, and a negative urinary myoglobin level clearly ruled out the possibility of neuroleptic malignant syndrome, malignant hyperthermia, lethal catatonia, and central anticholinergic syndrome.[9] Finally, the quick response to a serotonin receptor antagonist like cyproheptadine substantiates the diagnosis of serotonin syndrome. The antihistamine cyproheptadine, which is also a 5-HT_2A_ inhibitor can be considered in moderate and severe cases.[10,11]

## Conclusion

Few severe cases of serotonin syndrome have been reported due to concurrent use of MAOIs and SSRIs.[12] Serotonin syndrome has been found to have a mortality less than 1 per 1000 with a very low incidence. Fatality increases with increase in number of drugs taken together.[13] It can occur even with low doses of sertraline.[14] However, not many cases have been reported to be precipitated with therapeutic doses of escitalopram.[15,16] Moreover the present case suggests that even with great pharmacological precautions, there can be one or two such cases whose early diagnosis and prompt management can prove to be life saving for the patient.
